# Decoding the processing of lying using functional connectivity MRI

**DOI:** 10.1186/s12993-014-0046-4

**Published:** 2015-01-17

**Authors:** Weixiong Jiang, Huasheng Liu, Lingli Zeng, Jian Liao, Hui Shen, Aijing Luo, Dewen Hu, Wei Wang

**Affiliations:** Department of Radiology, The Third Xiangya Hospital, Central South University, Changsha, Hunan 410013 P.R. China; College of Mechatronics and Automation, National University of Defense Technology, Changsha, Hunan 410073 P.R. China; Department of Information Science and Engineering, Hunan First Normal University, Changsha, Hunan 410205 P.R. China; Key Laboratory of Medical Information Research (Central South University), College of Hunan Province, Changsha, Hunan 410083 P.R. China

**Keywords:** fMRI, Deception, Multivariate pattern analysis, Functional connectivity

## Abstract

**Background:**

Previous functional MRI (fMRI) studies have demonstrated group differences in brain activity between deceptive and honest responses. The functional connectivity network related to lie-telling remains largely uncharacterized.

**Methods:**

In this study, we designed a lie-telling experiment that emphasized strategy devising. Thirty-two subjects underwent fMRI while responding to questions in a truthful, inverse, or deceitful manner. For each subject, whole-brain functional connectivity networks were constructed from correlations among brain regions for the lie-telling and truth-telling conditions. Then, a multivariate pattern analysis approach was used to distinguish lie-telling from truth-telling based on the functional connectivity networks.

**Results:**

The classification results demonstrated that lie-telling could be differentiated from truth-telling with an accuracy of 82.81% (85.94% for lie-telling, 79.69% for truth-telling). The connectivities related to the fronto-parietal networks, cerebellum and cingulo-opercular networks are most discriminating, implying crucial roles for these three networks in the processing of deception.

**Conclusions:**

The current study may shed new light on the neural pattern of deception from a functional integration viewpoint.

**Electronic supplementary material:**

The online version of this article (doi:10.1186/s12993-014-0046-4) contains supplementary material, which is available to authorized users.

## Background

Deception is a rather complex mental activity. During lying, many functions of higher cognition are involved. Deception has been demonstrated to be associated with greater activation within the prefrontal cortex compared to truthfulness [1-6]. In our recent study on deception, increased neuronal activity was observed mainly in the dorsolateral prefrontal cortex during lie-telling compared to truth-telling, and as the capacity for deception increased, the contrast between brain activities (lie vs. truth) decreased [7]. Most of the previous studies of deception have focused on brain-activity patterns [1-4,6-17]. Over the past few years, functional connectivity has been widely used in the detection of brain dysfunction in neuropsychiatric diseases [18]. It remains unclear whether there are functional connectivity differences between lie-telling and truth-telling.

In recent years, interest in multivariate pattern analysis (MVPA) methods based on neuroimaging data has increased [19,20], perhaps because MVPA methods can provide unique information that is overlooked by univariate group-level statistical analysis approaches [21]. Davatzikos et al. used a high-dimensional non-linear pattern classification method to discriminate lie-telling from truth-telling based on brain activity [11], ignoring the interactions between brain regions (functional connectivity). Whole-brain functional connectivity patterns may provide a sensitive and informative signature of cognitive processing [22-25], so increased attention is being paid to examining large-scale functional connections during cognitive processing using MVPA methods [22-27]. We also succeeded in differentiating individuals with antisocial personality disorders from controls using resting-state functional connectivity in a recent study [28]. Here, we proposed that MVPA could extract an informative functional connectivity pattern to identify deception from truth-telling in an empirical study of deception.

To perform a specific task, humans are thought to enter a task-dependent cognitive state [29]. Humans have two separable and parallel control networks, i.e., fronto-parietal and cingulo-opercular networks, each of which exerts top-down control, though with different properties and over different temporal scales [30,31]. Whether and how the networks are modulated by deception is an interesting theme.

## Materials and methods

### Participants

We recruited 45 right-handed native Chinese speakers. The subjects had normal or corrected-to-normal vision; they had no history of substance abuse (e.g., alcohol or drugs) prior to the brain scan; and they had no major head trauma or a history or current diagnosis of serious mental disorders, e.g., depression, anxiety neurosis or schizophrenia, as assessed by two senior psychiatrists. The exclusion criteria after the MRI experiment were as follows: their response accuracies in the “true” and “inverse” conditions were lower than 80%; data with maximum displacement in any direction during scanning was greater than 2 mm or head rotations were larger than 2.0 degrees; or they had not devised strategies sufficiently well during lie-telling scanning. In 13 cases, the scan data were unsatisfactory (5 subjects had inadequate motivation for the lie condition, 3 subjects had movement-related artefacts that were identified post-scanning and 5 subjects had response accuracies during the true and inverse condition that were below 80%, as shown below). The final sample consisted of 32 volunteers (mean age = 20.20 ± 1.56 years).

After receiving a detailed description of the study, all volunteers provided written informed consent. Participants were paid a base rate of ¥100 for their participation, in addition to a ¥50 bonus based on their performance. This study was approved by the Ethical Committee of the Third Xiangya Hospital of Central South University.

### Experimental design and procedures

In this study, we used the picture choice task [7,32] to examine the brain network of deception. Each subject randomly extracted 3 pictures from 10 neutral pictures (e.g., a pair of shoes, an orange) prior to scanning, and then decided if the picture presented during scanning was one of the 3 items by following special instructions. These instructions comprised three conditions, i.e., a “true”, an “inverse” and a “lie” condition. During the true condition, the participants were required to give accurate, honest responses. During the inverse condition, the participants were required to give opposite responses; for example, when the picture presented was one of the 3 items chosen, the subject should press the “No” button and they were to press the “Yes” button otherwise. During the lie condition, the participants were required to devise a strategy to deceive others, with the goal of lying skillfully and avoiding detection. The subject could devise various strategies to decide when to respond honestly and when to respond falsely. For example, in the first lie block, he could respond in the manner of “TFTTFF” (T: true, F: false); in the second lie block, he could respond with another method of “FFTTTF”; in the third lie block, “FTFTFT”; and in the fourth lie block, “TTFFFT”. We added the “inverse” condition to indicate that lie-telling is not simply responding oppositely and requires devising a strategy.

**Figure 1:**
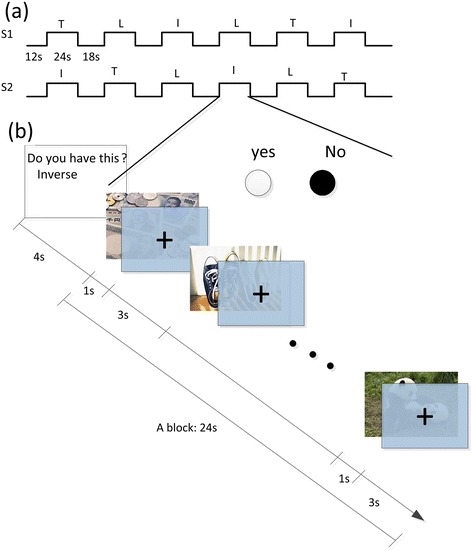
**The schematic diagram of the experiment: (a) Pseudorandom order of two sequences.** T = true, I = inverse, L = lie. **(b)** Schematic representation of a block.

A block design consisting of two sequences was used in this study. During scanning, the 10 pictures appeared randomly, the probability of occurrence of each picture was approximately equal in each sequence, and each picture appeared, at most, one time in each block. Each sequence lasted 264 -s and contained six blocks. The six blocks were presented in a pseudorandom order (Figure 1a), in which two blocks required true responses, two required inverse responses and two required lie responses. Each sequence began with an 8 -s rest period. Specific instructions regarding the response types were presented for 4 -s before each block began. Each block lasted for 24 -s. The rest time that followed each block lasted for 14 -s (the final rest time for each sequence was 18 -s, Figure 1a).

Each block consisted of six picture stimuli. Each stimulus was presented for 1 -s and was followed by a visual fixation crosshair for 3 -s. The participants were instructed to indicate whether the presented picture was one of the 3 items that they had chosen beforehand by pressing the “yes” or “no” button, according to the specified instructions. At the end of the fixation, a fresh stimulus was presented for 1 -s, followed by a 3 -s fixation (Figure 1b).

At the beginning of the experiment, the participants underwent a training session, the block order of which was different from that during scanning, to familiarize the participants with the test and the hardware outside the scanner. During the post-experimental debriefing that followed the scanning, we first asked what stratagem they had used in each lie condition, and then asked them to indicate their degree of confidence that they had deceived someone else on a Likert scale from 0 to 3, with 3 indicating the highest level of confidence and 0 the lowest. This procedure was intended to check the degree of the subjects’ motivations to lie. A total of 40 subjects rated themselves as 2 or higher, and 5 subjects were excluded because they rated themselves as 1 or lower.

### Image acquisition and pre-processing

Functional images were acquired on a 1.5 Tesla Siemens Trio scanner at the Third Xiangya Hospital of Central South University using T2*-weighted echo planar imaging with a blood-oxygen-level-dependent (BOLD) contrast pulse sequence (23 slices, thickness/gap = 5.0/1.2 mm, matrix = 64 × 64, repetition time = 2000 ms, echo time = 50 ms, flip angle = 90°, and field of view = 240 mm × 240 mm).

Functional data were pre-processed using a statistical parametric mapping software package (SPM8, www.fil.ion.ucl.ac.uk/spm). For each sequence, we discarded the first 6 volumes to allow for T1 equilibration effects, leaving 126 volumes for further analysis. All datasets were corrected for temporal offsets and for head movement-related effects. Data with a maximum displacement in any direction greater than 2 mm or head rotations larger than 2.0 degrees were excluded. The resulting datasets were spatially transformed and resampled to a standardized brain (Montreal Neurologic Institute, 2-mm isotropic voxels) and then smoothed with an 8-mm full-width half-maximum Gaussian kernel.

### Behavioral data analysis

To ensure cooperation, we calculated the response accuracy and response time for each participant and excluded 5 subjects whose response accuracies in the true and inverse conditions were lower than 80%. In the true condition, accuracy was calculated according to whether the participants gave accurate and honest responses. For example, when the picture was one of the 3 items that the subject had chosen at the beginning, the subject was to press the “Yes” button, and they were to press the “No” button otherwise. In the inverse condition, accuracy was calculated according to whether the participants gave opposite responses; for example, when the picture was one of the 3 items that the subject had chosen, the subject was to press the “No” button, and they were to press the “Yes” button otherwise. In the lie condition, accuracy was calculated against ground-truth. The items selected by each participant were collected by the researcher before the scan session.

To investigate whether response accuracy and response time were related to response type, two-way repeated measures ANOVAs were employed.

### Functional connectivity networks for truth and lie processing

We first obtained time series of the lie and true conditions. In this step, we made a regression for head motion to account for motion artefacts, used a high-pass filter of 1/128 Hz to remove low-frequency noise, and then segmented and concatenated the time series in terms of the lie and truth conditions individually.

As implemented by Dosenbach et al. [30], we functionally defined nodes using the same 160 MNI coordinates and trimmed them to ensure no overlap with each other. These nodes were selected and defined based on separate meta-analyses of task-related fMRI studies. The distance between all ROI (regions of interest) centers was at least 10 mm. For a more detailed description, see [30]. All time-series across the same condition were divided into the 160 regions. For each subject, the time-series for each region were extracted using singular value decomposition (SVD) and we retained the top 2 eigenvariates from each region. According to studies by Pantazatos et al. [22,23], compared with the mean signal in each region, SVD helps to avoid inadvertently ignoring important variation within regions or averaging them away. Nuisance covariates, including the global mean and white matter and cerebrospinal fluid signals, were removed from the time series of each condition prior to time-series extraction of ROI [28,33-35]. For each eigenvariate, Pearson’s correlation coefficients were used to evaluate the functional connectivity between each pair of regions, and we obtained two condition-dependent functional networks for each subject that were expressed as a 160 × 160 symmetrical matrix for each condition. By removing the 160 diagonal elements, the 12720 upper triangular elements of the functional connectivity matrix were normalized using Fisher’s z-transformation [36] and then used as the features for all subsequent multivariate pattern analyses.

### Pattern analysis of functional connectivity to distinguish lie-telling from truth-telling

When classification features were obtained, support vector machines (SVM) were employed to solve the classification problem [37,38]. An SVM classifier aims to find a hyperplane maximizing the margin between positive and negative samples while simultaneously minimizing misclassification errors in the training set [39]. In this study, linear SVMs were used together with filtering feature selection based on the Kendall tau rank correlation coefficient [28,40] and leave-two-out cross validation (LTOCV). There were 64 examples for each condition (2 eigenvariates from each condition, 32 subjects in total). During each iteration of 64 rounds of LTOCV, both examples (2 eigenvariates from the same condition of the same subject) from one condition were withheld from the dataset. Subsequently: 1) the Kendall tau rank correlation coefficient was calculated over the remaining training data; 2) the features were ranked by absolute tau- score and the top N were selected; and 3) these selected features were used to train the classifier and predict the categories of the withheld test examples. Finally, we calculated the classification accuracy. To determine the optimal number of the selected features, we repeated the classification with a varying number of features that were ranked by their tau- scores.

To assess the statistical significance of the LTOCV results, we used permutation tests [41,42]. The training data labels were randomly permuted 10,000 times for permutation testing. We then performed LTOCV on every permuted training set and defined GR0 as the generalization rate obtained by the classifier trained on the real class labels. When GR0 exceeded the 95% (P < 0.05) confidence interval of the classifier trained on randomly re-labeled class labels, the classifier was proposed to have reliably learned the relationship between the data and the labels. The P-value showed the probability of observing a classification prediction rate of no less than GR0.

## Results

### Behavioral data

In the true, inverse, and lie conditions, the response accuracies of the 32 subjects were (94.56 ± 4.45%), (87.67 ± 6.36%), and (44.37 ± 11.56%), respectively, and the reaction times were (0.68 ± 0.27 s), (0.85 ± 0.34 s), and (0.95 ± 0.45 s), respectively. We found significant effects of response type, characterized by longer reaction times (P < 0.0001) and reduced response accuracies (P < 0.0001) in the lie task, indicating that subjects performed the lie task appropriately.

### Classification results

**Figure 2:**
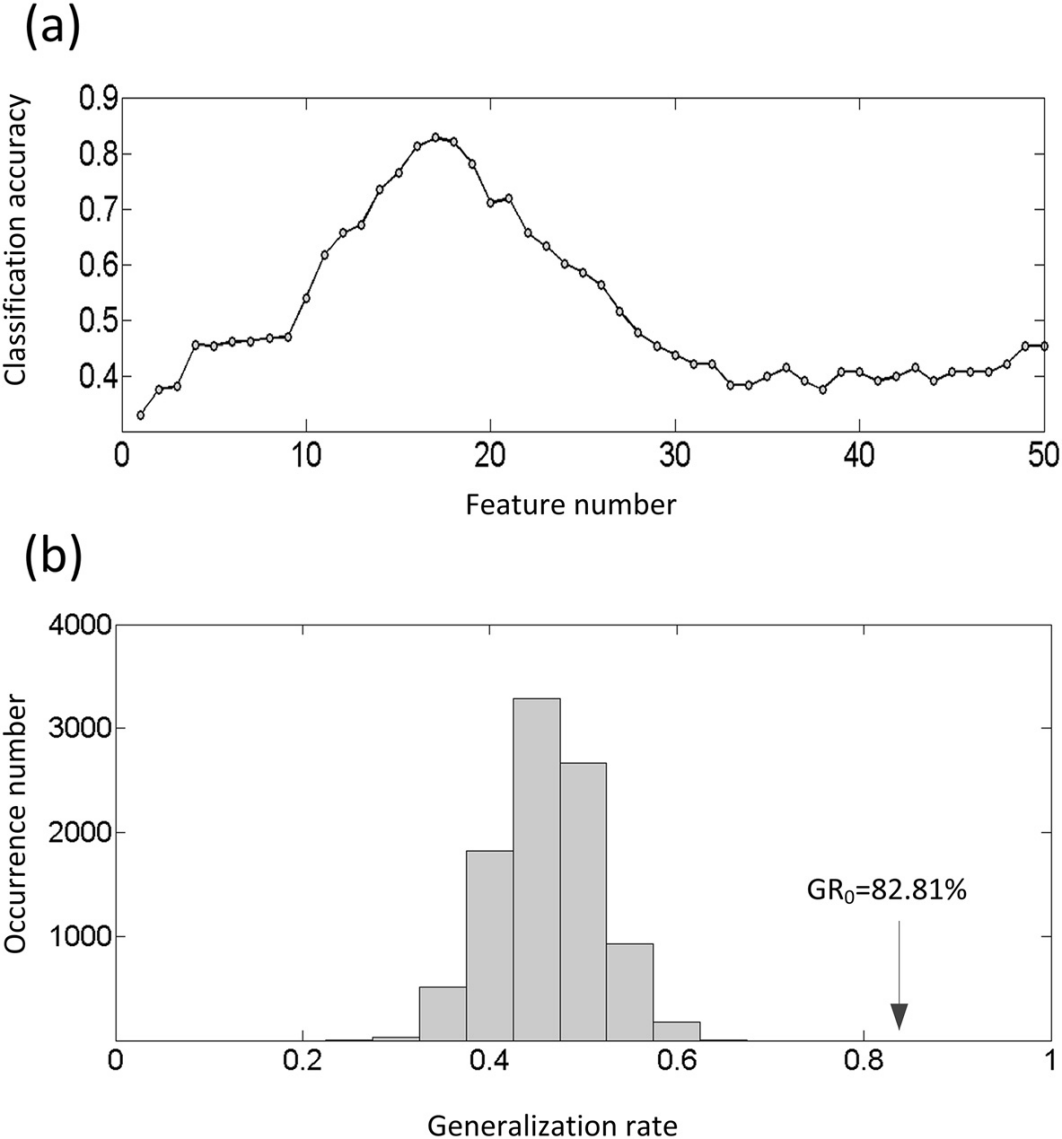
**Performance evaluation of the classifier. (a)** The curve of the generalization rate to the number of features. **(b)** Permutation distribution of the estimate (repetition times: 10,000). GR0 is the generation rate obtained by the classifier trained on the real class labels. With the generalization rate statistic, this figure reveals that the classifier learned the relationship between the data and the labels with a <0.0001 probability of being wrong.

To determine the optimal size of the feature subset, we repeated the classification with a varying number of different features and found that the classifier's best performance was achieved when selecting the 17 most discriminating functional connections (Figure 2a). The classification accuracy was 82.81% (85.94% for the lie condition, 79.69% for the true condition) via LTOCV. Permutation tests revealed that the proposed classifier learned the relationship between the data and the labels with a less than 0.0001 risk of being wrong (P < 0.0001, Figure 2b).

With this in mind, we performed multiple tests. When extracting only one eigenvariate per region, the maximum accuracy was 62.5% for the 1st eigenvariate when 25 functional connection feathers were used and 54.69% for the 2nd eigenvariate when 36 functional connection feathers were used. This may have occurred because larger regions encompassed other functional subregions that were not included in the analysis. Another possible reason is that for many regions, the 1st eigenvariate may reflect variation caused by other conditions/blocks within the run that were not considered in the current classification analyses, or it could be a combination of all of the above[22,23]. When we used 320 × 320 matrices resulting from two principal components per node, the maximum accuracy was 62.5% when 19 functional connection feathers were used. This method may have utilized serial information. When we split the top 2 eigenvariates from each node into separate examples, we may have better utilized the correlate information between the two component networks.

### Functional connectivity pattern of lie-telling differs from that of truth-telling

**Figure 3:**
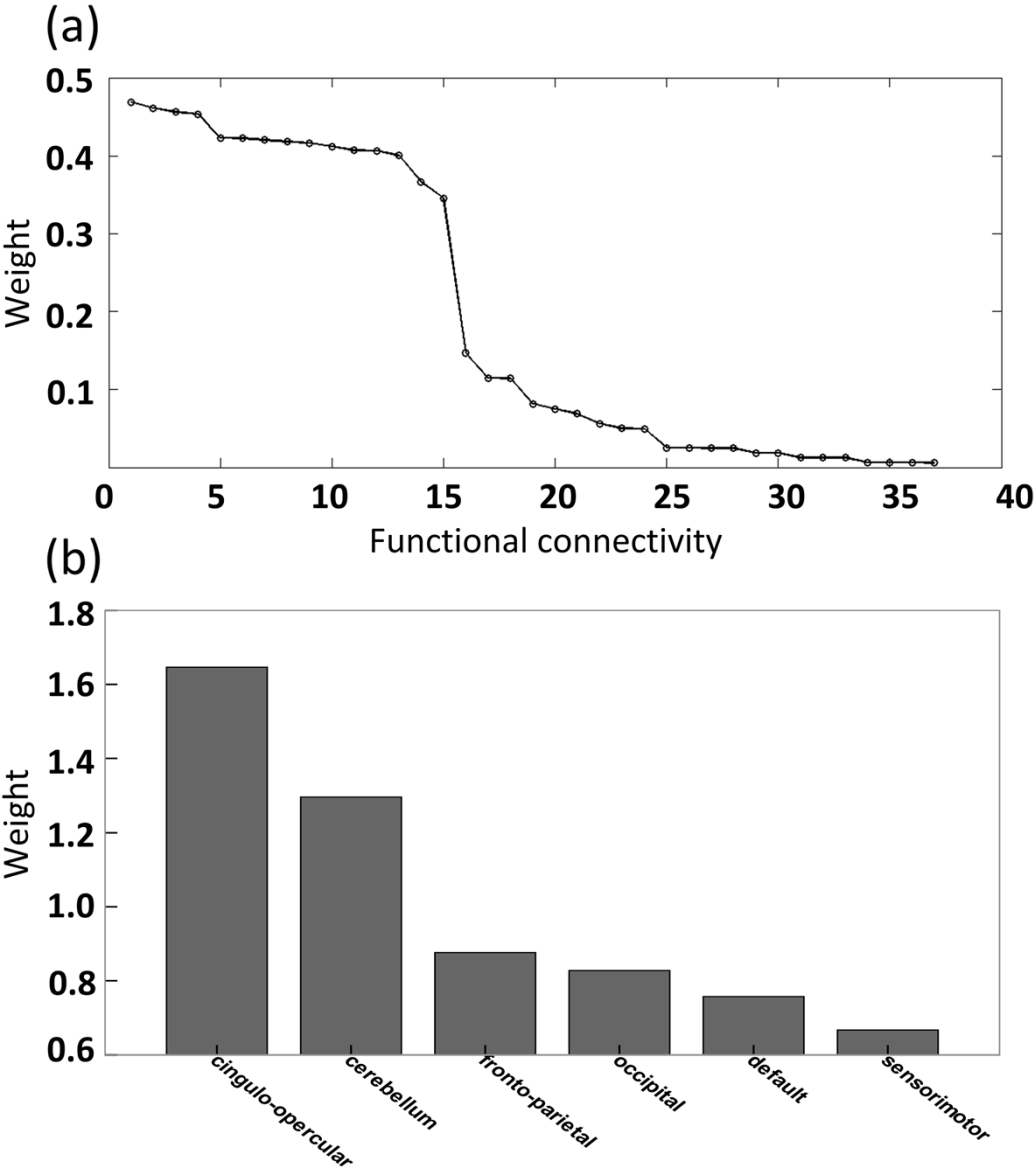
**Characteristic analyses of deception-modulated functional connectivity. (a)** τ value distribution of all 37 features represented in the LTOCV. The horizontal axis represents each functional connection and the vertical axis represents the weighted Kendall tau correlation coefficient. **(b)** Summarized weights for each of the six communities.

The selected functional connectivity feature set may be slightly different in each iteration of LTOCV. A total of 37 features emerged during LTOCV when selecting the 17 most discriminating functional connections. The discriminative power of each feature was computed by multiplying the mean Kendall tau correlation coefficient by the occurrence rate across all iterations of cross-validation [28], and the 15 features that appeared in no fewer than 52 iterations were found to have the largest discriminative power and could best distinguish the two conditions (Figure 3a). These highly discriminating functional connections represented condition-modulated functional connectivity patterns, including 10 stronger connections that exhibited positive modulation by deception and 5 weaker connections that exhibited negative modulation by deception (Table 1). The results of two-tailed two-sample t-tests revealed that the 15 most discriminating functional connections were significantly different between lie-telling and truth-telling (P < < 0.001).

**Table 1:** **Deception-modulated functional connections**

**Modulated features**	**MNI coordinates**	**Length (mm)**	**τ value**	**P value**
	**( x, y, z )**			
**Increased connections (lie > true)**				
aPFC/inf_cerebellum	(29, 57, 18)/(−34, −67, −29)	147	0.4688	3.1675E-06
vFC/post_occipital	(43, 1, 12)/(27, −91, 2)	94	0.4609	3.7259E-06
vIPFC/inf_cerebellum	(39, 42, 16)/(−34, −67, −29)	139	0.4233	5.5833E-05
dACC/occipital	( 9, 20, 34)/(20, −78, −2)	105	0.4204	2.6348E-05
post_insula/thalamus	(−30, −28, 9)/(11, −12, 6)	44	0.4121	1.3363E-04
vPFC/post_occipital	(34, 32, 7)/(29, −81, 14)	113	0.4185	1.1820E-04
aPFC/sup_parietal	(27, 49, 26)/(34, −39, 65)	97	0.4165	1.7691E-05
vFC/temporal	(−48, 6, 1)/(43, −43, 8)	104	0.4064	1.0832E-04
IPL/occipital	(−53, −50, 39)/(−44, −63, −7)	49	0.4070	5.2067E-05
IPS/inf_temporal	(−36, −49, 60)/(−61, −41, 2)	64	0.3454	5.7618E-05
**Decreased connections (lie < true)**				
vFC/med_cerebellum	(5, −75, −11)/(43, 1, 12)	88	−0.4561	1.7871E-05
IPL/inf_cerebellum	(−48, −47, 49)/(−6, −79, −33)	98	−0.4531	2.0318E-06
lat_cerebellum/occipital	(−24, −44, −25)/(45, −72, 29)	100	−0.4229	1.3987E-04
vIPFC/temporal	(46, 39, −15)/(43, −43, 8)	85	−0.4007	3.3076E-05
inf_cerebellum/post_occipital	(18, −81, −33)/(27, −91, 2)	37	−0.3667	5.7618E-05

**Figure 4:**
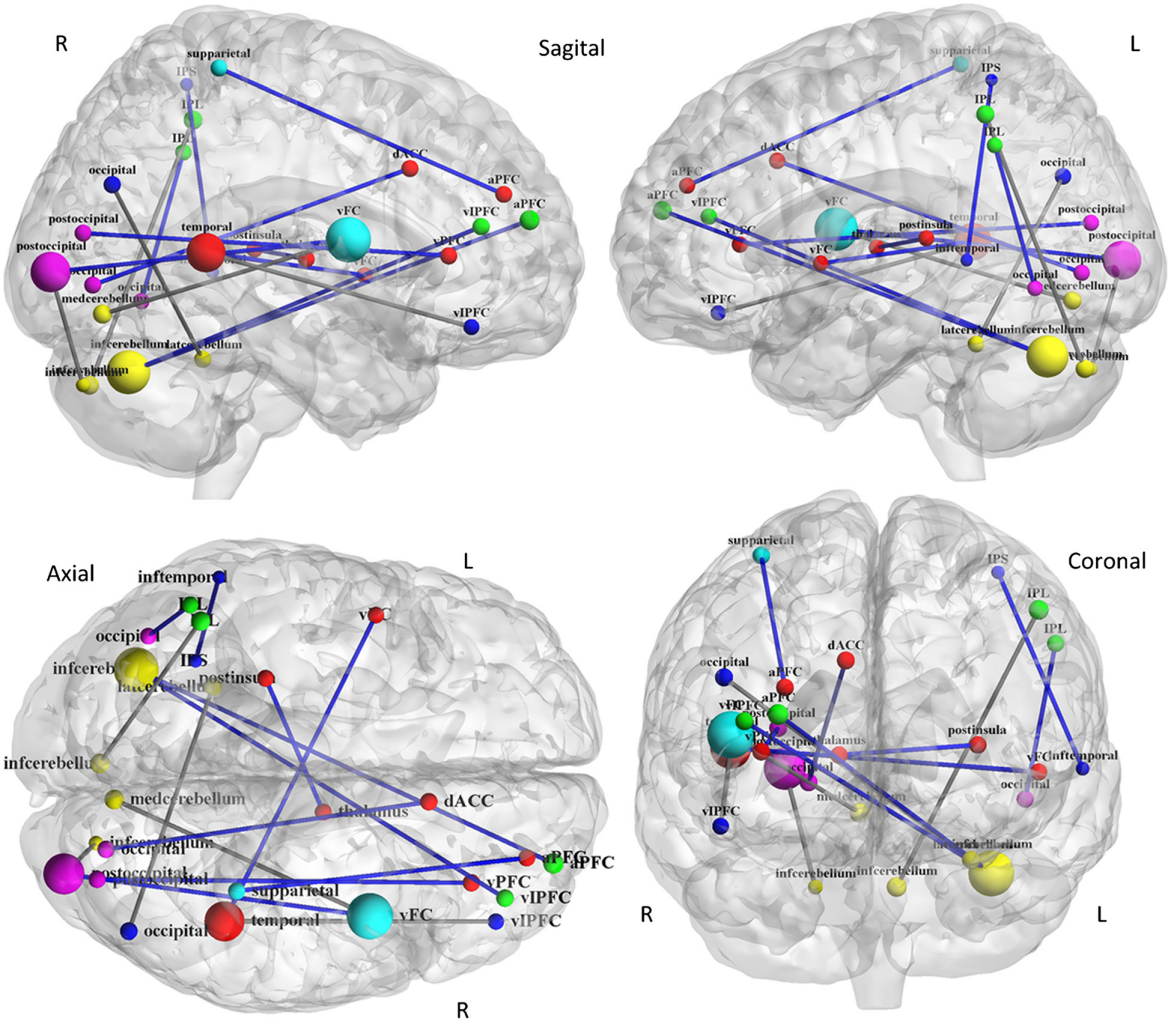
**Deception-modulated functional connectivity.** Regions are color-coded by category. Red represents the cingulo-opercular network; green represents the fronto-parietal network; dark blue represents the default network; light blue represents the sensorimotor network; purple presents the occipital network; and yellow represents the cerebellum. Functional connectivities are also color-coded, with blue lines representing stronger connections and gray lines representing weaker connections.

One of the most intriguing findings here was that these modulated functional connections were mostly spatially remote (i.e., long-range; length > 80 mm; Figure 4, Table 1). These analyses revealed that longer connections contributed more to deception (73.3%) than did shorter connections (26.7%). The 15 functional connections with high discriminative power connected 26 brain regions. According to a canonical template of resting-state networks [30], the 26 brain regions were grouped into six networks: cingulo-opercular, fronto-parietal, default, sensorimotor, occipital and cerebellar. Summing the feature weights for each network, the results revealed that the cingulo-opercular control network had the greatest sum total of feature weights (Figure 3b, Table 2), suggesting that they were the best relative predictors. In addition, the cerebellar and fronto-parietal control networks had large weights. Another finding was that these deception-modulated connections were mostly between networks (80%; Table 2). We also used leave-four-out cross validation (LFOCV) when decoding the processing of lying, i.e. four examples from one subject were withheld from the dataset. For the relevant results, please see the Additional file 1.

**Table 2:** **Deception-modulated network**

**Network**	**Total**	**Within networks**	**Between networks (absolute)**
Cingulo-opercular	1.6466	0.8186	0.828
Cerebellum	1.2954	0	1.2954
fronto-parietal	0.8761	0	0.8761
Occipital	0.8275	0	0.8275
Default	0.7572	0.3454	0.4118
Sensorimotor	0.6667	0	0.6667

## Discussion

In this study, we imitated deception that emphasized strategy devising. The results demonstrated that the functional connectivity pattern provided sufficient information to decode deception. The most discriminating functional connections included 10 stronger ones with positive modulation by deception and 5 weaker ones with negative modulation by deception, which were mostly spatially remote and located between networks. The connectivities related to the cingulo-opercular, cerebellar and fronto-parietal networks are most discriminating, implying crucial roles for these three networks in the processing of deception.

### A method to estimate deception-modulated functional connectivity

Recent fMRI studies have characterized deception from the brain activity patterns but have ignored the functional connectivity between brain regions. In the current study, a multivariate pattern analysis with whole-brain functional connectivity revealed that whole-brain functional connectivity patterns are able to differentiate lie-telling from truth-telling with a promising accuracy of 82.81% (85.94% for lie-telling, 79.69% for truth-telling), suggesting that whole-brain functional connectivity could provide a sensitive and informative signature for decoding the processing of deception at the individual level and complementing univariate statistical analyses. Analyses of functional connectivity could shed new light on the neuronal patterns of deception, further indicating that brain conditions may not only be tied to the activity of a set of brain areas but also to the way the regions are connected functionally [22,23,43-45].

### Deception-modulated network

Humans are thought to enter a task-dependent cognitive state when performing a specific task [29]. Humans have two separable and parallel control networks, i.e., fronto-parietal and cingulo-opercular networks [30,31]. In our current study, the connectivity related to the fronto-parietal networks, cingulo-opercular networks and cerebellum exhibited the highest discriminative power. When performing lie-telling tasks, the subjects may adopt task sets that flexibly configure information processing under the control of the two networks in response to changing task demands.

In this study, the connection between the dACC and occipital cortex was found to strengthen during deception. Abe et al. [8] found that the ACC was activated when deceptively responding to previously experienced stimuli. The ACC may monitor the reading of and deceptive responding to a stimulus [10]. The ACC has been implicated in neurobiological models of cognitive control, the inhibition of competing or prepotent responses, the mediation of conflict, and reward and motivation [46,47], particularly in decision making [48-50]. It has also been suggested that the dACC/msFC may form part of an attention or executive control system [51-53]. It stands to reason that the connection between the dACC/msFC and the occipital cortex may carry out functions that are most central to the implementation of deception.

Some connections related to the prefrontal cortex (PFC) region were found to exhibit high discriminative power. Previous neuro-imaging studies have shown that the PFC plays a significant role in deception [5,54,55]. Many studies have suggested that the anterior PFC (aPFC) maintains task and context information [56-58]. The aPFC may provide more specific representations of plans, subgoals, rules [59,60], and/or strategies [61] for complex task paradigms. The aPFC has been associated with complex higher-order task-control functions [58,62]. In our study, the connectivities between the aPFC and the inferior cerebellum and the post occipital cortex may be in response to high demands on working memory and control by deception.

Some previous studies have suggested that the connection between the thalamus and the insula played a prominent role during implicit emotion perception [22,23]. We speculate that such functional connectivity may be related to the emotional conflict caused by lie-telling. The IPS is thought to play a major role in the top– down control of attention [63]. Dosenbach et al. proposed that the IPS occupies a central integrative position in the fronto-parietal network that exerts top–down control [64]. The connection between the IPS and the inferior temporal gyrus may help load, transmit, or instantiate the required task-set parameters at the beginning of each task period. The IPL is sensitive to conflict at the level of stimulus presentation [65] and the cerebellum may generate error codes [66]. The connection between the IPL and cerebellar regions suggests that the IPL may receive cerebellar error signals that support the rapid, continual fine-tuning of control settings and decision making.

The finding of connections related to cerebellar regions was not surprising: although the main function of the cerebellum is motor processing, previous studies have underlined the importance of the cerebellum in various cognitive domains, including executive functions [67,68], working memory [69-72], and attention [73,74]. These functions of the cerebellum are crucial for deception. The cerebellum has also been identified as involved in processing error codes [30,31,75,76]. The cerebellum may send error codes to both control networks and/or it may receive error information from one or both of the control networks of the brain. The cerebro-cerebellar circuits may underlie the involvement of the cerebellum in deception.

To successfully avoid detection, the deceiver must calculate the odds of being detected, remember the previous responses given, inhibit the normal propensity towards truth-telling, and then choose an appropriate strategy prior to making a response. As such, it is possible that the deception-modulated network and functional connections are the result of the complex interplay of working memory, response inhibition, sustained attention, mental calculations and execution that is necessary for our subjects to make deceptive responses. Greater cognitive load, as an important and integrated feature characterizing lying, may consist of these components. This greater cognitive load during lying was also reflected in reaction times as mentioned previously. In the current study, the varying functional connections may reflect the greater cognitive load, and the classification may be picking up differences in cognitive load and processing time-on-task.

### Long-range characteristic of deception-modulated connectivity

Long-range connectivity is important for the functional–anatomical organization of the human brain [77]. Long-range functional connections tend to increase over time, indicating that long-range functional connections are related to intelligence development [30,78]. Long-range connectivity has also been suggested to be related to higher cognitive functions [79]. In our study, the increases in long-range connectivity modulated by deception were interesting, and may have occurred in response to increased cognition or increased intelligent demand during lie-telling.

The present study demonstrated that deception could be classified using whole-brain functional connectivity MRI. The identified discriminating functional connectivity may shed new light on the neural pattern of lie-telling from a functional integration viewpoint. The main findings will be confirmed with a larger independent dataset in the future. There are some limitations to the task design. Deception in the current experiment was not an authentic representation of deception in real-life situations. The block design of deception experiments has limited external validity. In our future studies, more valid designs will be considered, e.g., an event-related design [80-83]. However, varying functional connections may reflect the greater cognitive load, and the classification may be picking up differences in cognitive load and processing time-on-task. Therefore, we will study lying from this perspective in the future.

## Additional file

Additional file 1:
**Supplement Materials.**
