# Supplementary material for: Decoding the processing of lying using functional connectivity MRI
Source: Behav Brain Funct. 2015 Jan 17;11(1):1. doi: 10.1186/s12993-014-0046-4 (PMC4316800; doi:10.1186/s12993-014-0046-4)
Supplement: Additional file 1: — Supplement Materials. [file 12993_2014_46_MOESM1_ESM.doc]

**Supplement Materials**

# Decoding the processing of lying using functional connectivity MRI

Weixiong Jiang^1,3^*, Huasheng Liu^1^*, Lingli Zeng^2^, Jian Liao^1^, Hui Shen^2^, Aijing Luo^4^**,** Dewen Hu^2§^, Wei Wang^1,4§^

# The results of leave-four-out cross validation (LFOCV)

We also used leave-four-out cross validation (LFOCV) when decoding the processing of lying using functional connectivity MRI. During each iteration of 32 rounds of LFOCV, four examples from one subject were withheld from the dataset. Subsequently: 1) the Kendall tau rank correlation coefficient was calculated over the remaining training data; 2) the features were ranked by absolute tau- score and the top N were selected; and 3) these selected features were used to train the classifier and predict the categories of the withheld test examples. Finally, we calculated the classification accuracy. To determine the optimal number of the selected features, we repeated the classification with a varying number of features that were ranked by their tau- scores. The classifier's best performance was achieved 75.09% when selecting the 21 most discriminating functional connections (Figure S1).

The selected functional connectivity feature set may be slightly different in each iteration of LFOCV. A total of 45 features emerged during LTOCV when selecting the 21 most discriminating functional connections. The discriminative power of each feature was computed by multiplying the mean Kendall tau correlation coefficient by the occurrence rate across all iterations of cross-validation, and the 18 features that appeared in no fewer than 24 iterations were found to have the largest discriminative power and could best distinguish the two conditions (Table S1, Figure S2). These highly discriminating functional connections represented condition-modulated functional connectivity patterns, including 12 stronger connections that exhibited positive modulation by deception and 6 weaker connections that exhibited negative modulation by deception (Table S1). The results of two-tailed two-sample t-tests revealed that the 18 most discriminating functional connections were significantly different between lie-telling and truth-telling (P << 0.001). This analysis using LFOCV is likely to be less biased than the LTOCV in the main text.


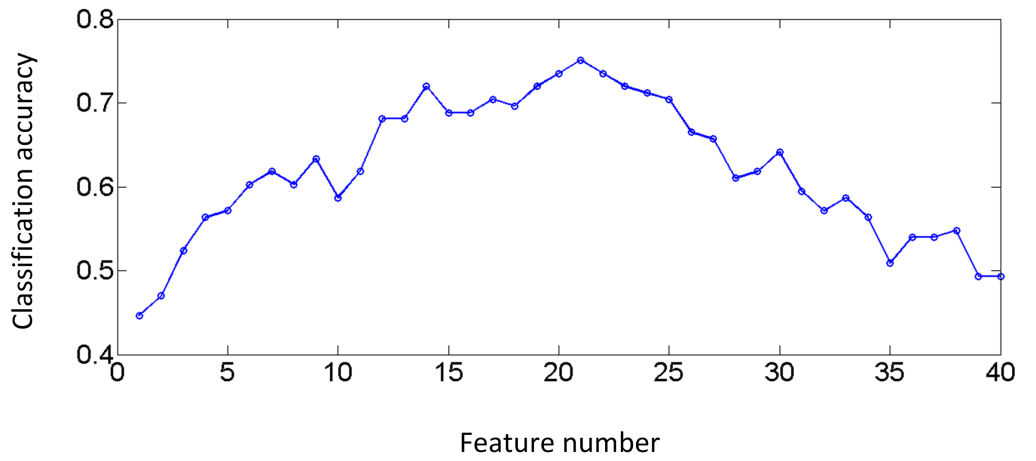


Figure S1 The curve of the generalization rate to the number of features under LFOCV

Table S1 Deception-modulated functional connections under LFOCV

| Modulated features | MNI coordinates | Length (mm) | τ value | ttest |
| --- | --- | --- | --- | --- |
|  | ( x, y, z ) |  |  | P value |
| **increasing connections (lie>true)** | |  |  |  |
| dACC/occipital | ( 9, 20, 34) / ( 20, -78, -2) | 105 | 0.4561 | 2.6348E-05 |
| TPJ/ant_insula | (-52, -63, 15)/(38, 21, -1) | 124 | 0.4532 | 3.3240E-06 |
| aPFC/inf_cerebellum | ( 29, 57, 18) / (-34, -67, -29) | 147 | 0.4235 | 3.1675E-06 |
| vIPFC / inf_cerebellum | ( 39, 42, 16) / (-34, -67, -29) | 139 | 0.4229 | 5.5833E-05 |
| vFC/post_occipital | ( 43, 1, 12) / ( 27, -91, 2) | 94 | 0.4204 | 3.7259E-06 |
| IPL/dIPFC | (-48, 47, 49)/(46, 28, 31) | 98 | 0.4160 | 3.5423E-05 |
| basal_ganglia1/inf_cerebellum | (-6, 17, 34)/(32, -61, 31) | 87 | 0.4134 | 3.7930E-05 |
| vPFC / post_occipital | ( 34, 32, 7) / ( 29, -81, 14) | 113 | 0.4131 | 1.1820E-04 |
| fdIPFC/inf_cerebellum | (46, 28, 31)/(-34, -67, -29) | 133 | 0.4110 | 5.2169E-05 |
| vFC / temporal | (-48, 6, 1) / ( 43, -43, 8) | 104 | 0.4100 | 1.0832E-04 |
| post_insula/thalamus | (-30, -28, 9) / ( 11, -12, 6) | 44 | 0.4068 | 1.3363E-04 |
| aPFC/sup_parietal | ( 27, 49, 26) / ( 34, -39, 65) | 97 | 0.4043 | 1.7691E-05 |
|  |  |  |  |  |
| **decreasing connections (lie<true)** | |  |  |  |
| IPL/inf_cerebellum | (-48, -47, 49) / ( -6, -79, -33) | 98 | -0.4538 | 2.0318E-06 |
| angular_gyrus/occipital | (-41, -47, 29)/(9, -76, 14) | 60 | -0.4460 | 3.2036E-06 |
| vIPFC / temporal | ( 46, 39, -15) / ( 43, -43, 8) | 85 | -0.4084 | 3.3076E-05 |
| vFC / med_cerebellum | ( 5, -75, -11) / ( 43, 1, 12) | 88 | -0.4044 | 1.7871E-05 |
| med_cerebellum/occipital | (1, -66, -24)/(20, -78, -2) | 33 | -0.4035 | 3.2510E-04 |
| lat_cerebellum/occipital | (-24, -44, -25) / ( 45, -72, 29) | 100 | -0.4034 | 1.3987E-04 |


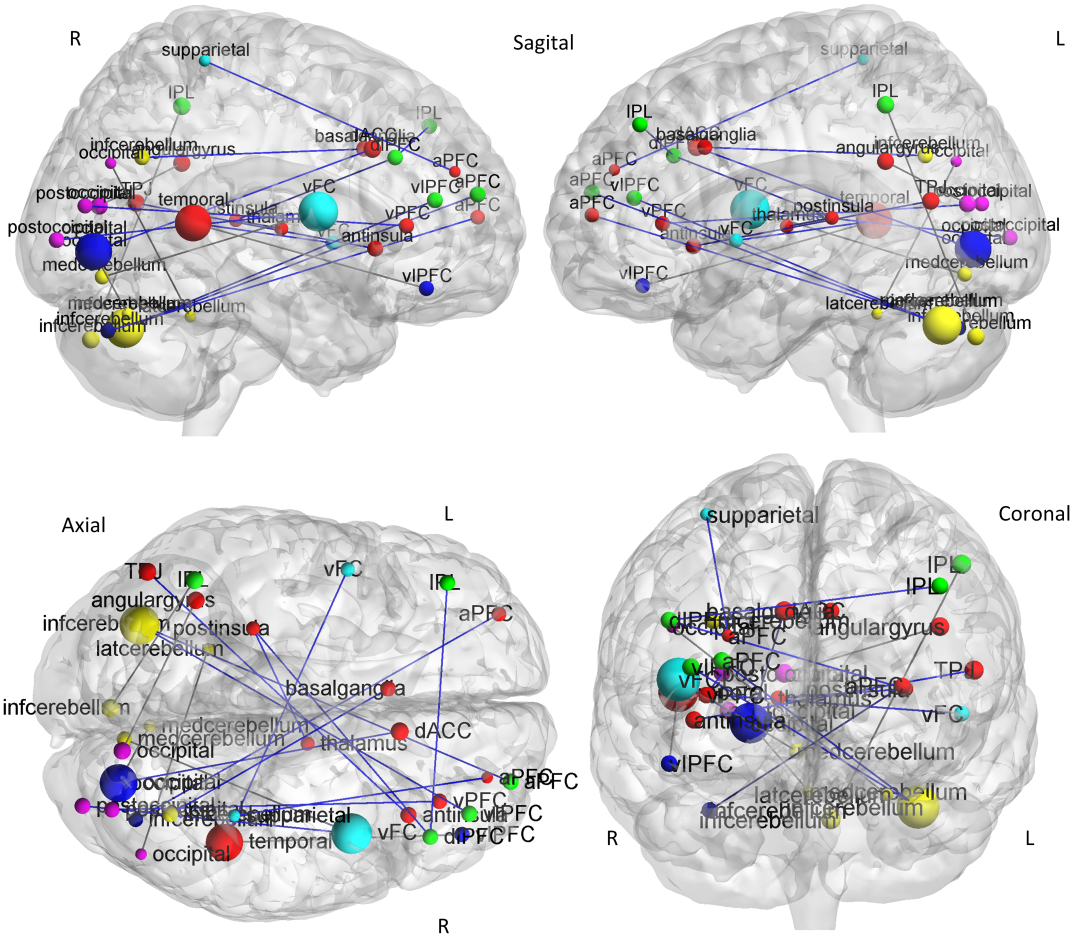


Figure S2 Deception-modulated functional connections under LFOCV
